# Root cap-dependent gravitropic U-turn of maize root requires light-induced auxin biosynthesis via the YUC pathway in the root apex

**DOI:** 10.1093/jxb/erw232

**Published:** 2016-06-15

**Authors:** Hiromi Suzuki, Ken Yokawa, Sayuri Nakano, Yuriko Yoshida, Isabelle Fabrissin, Takashi Okamoto, František Baluška, Tomokazu Koshiba

**Affiliations:** ^1^Department of Biological Sciences, Tokyo Metropolitan University, Tokyo 192-0397, Japan; ^2^IZMB, University of Bonn, D-53115 Bonn, Germany

**Keywords:** Indole-3-acetic acid (IAA) biosynthesis, light dependent, maize, root cap, root gravitropism, yucasin, *Zmvt2*, *Zmyuc*.

## Abstract

Gravitropism refers to the growth or movement of plants that is influenced by gravity. Roots exhibit positive gravitropism, and the root cap is thought to be the gravity-sensing site. In some plants, the root cap requires light irradiation for positive gravitropic responses. However, the mechanisms regulating this phenomenon are unknown. We herein report that maize roots exposed to white light continuously for ≥1–2h show increased indole-3-acetic acid (IAA) levels in the root tips, especially in the transition zone (1–3mm from the tip). Treatment with IAA biosynthesis inhibitors yucasin and l-kynurenine prevented any increases in IAA content and root curvature under light conditions. Analyses of the incorporation of a stable isotope label from tryptophan into IAA revealed that some of the IAA in roots was synthesized in the root apex. Furthermore, *Zmvt2* and *Zmyuc* gene transcripts were detected in the root apex. One of the *Zmyuc* genes (*ZM2G141383*) was up-regulated by light irradiation in the 0–1mm tip region. Our findings suggest that IAA accumulation in the transition zone is due to light-induced activation of *Zmyuc* gene expression in the 0–1mm root apex region. Light-induced changes in IAA levels and distributions mediate the maize root gravitropic U-turn.

## Highlight

The light-dependent gravitropic U-turn behaviour of maize roots is based on a *de novo* YUC pathway-dependent increase in auxin abundance in the root apex transition zone

## Introduction

Plants are continually exposed to many environmental stimuli. Gravity is a stimulus that causes roots to grow downward and shoots to grow upward. Charles and Francis Darwin studied root thigmotropism and nutation in several plants, mainly *Vicia faba* and *Pisum sativum*, and determined that the root tip (i.e. the root cap) was essential for root movement and gravity sensing (Darwin and Darwin, 1880; Chapters III and XI). They proposed that the tip region was important for the perception of stimuli such as touch, water, or gravity, and that ‘some influence’ moved from the tip to the growing region. They and other researchers observed that the removal of the *Zea mays* root cap impaired the gravitropic response, but it did not reduce the root growth rate (Ciesielski, 1872; Darwin and Darwin, 1880; Juniper *et al.*, 1966). Further research on maize roots indicated that the root cap is the source of water-soluble compounds responsible for mediating gravitropism (Pilet, 1971). Subsequent experiments revealed that a *Z. mays* mutant whose roots had little or no mucilage lacked a gravitropic response under white light when its root cap was touched only at its extreme apex (Moore *et al.*, 1990). These results suggested that the movement of a gravitropism effector from the root cap to the root may be necessary for a normal gravitropic response in *Z. mays*.

Genetic investigations on gravitropism have focused mainly on *Arabidopsis thaliana*. It is very difficult to detach the Arabidopsis root cap surgically; however, the importance of the root cap for a gravitropic response has previously been determined by removing or disrupting the root cap using a laser (Blancaflor *et al.*, 1998), by genetic ablation (Tsugeki and Fedoroff, 1999), or with a heavy-ion microbeam (Tanaka *et al.*, 2002). These experiments revealed that the columella cells in the root cap are the site of gravity perception. Amyloplasts containing starch in columella cells were proposed to act as statoliths in sensing gravity (Morita, 2010; Sato *et al.*, 2015).

In plants, the major form of the hormone auxin, indole-3-acetic acid (IAA), is thought to play an essential role in plant development. It is generally accepted that, in the coleoptile of monocots such as maize, IAA synthesized in the tip region is transported downward, causing the tip to curve as a result of asymmetrical growth in the lower region (Nishimura *et al.*, 2009). It was postulated that IAA is involved in inducing root and coleoptile curvature, and that the substance referred to as ‘some influence’ by the Darwins (1880) is in fact IAA. In practice, many experiments have revealed that when the root apex senses gravity, auxin is redistributed such that there are higher concentrations on the lower side of roots, and lower concentrations on the upper side of roots, with respect to the direction of gravity (Kleine-Vehn *et al.*, 2010; Sato *et al.*, 2015). Experiments with [^3^H]IAA have indicated that the root cap is not only the site of gravity perception but also the site of the initial redistribution of IAA in maize (Young *et al.*, 1990). Feldman (1980) reported that isolated maize roots could produce [^14^C]IAA from [^14^C]tryptophan, suggesting that the root cap is necessary for IAA metabolism. Furthermore, in Arabidopsis, studies have shown an asymmetric IAA distribution from the columella cells to the lateral root cap (Ottenschläger *et al.*, 2003), as well as differential IAA accumulation between the lower and upper sides of roots against gravity (Band *et al.*, 2012; Brunoud *et al.*, 2012; Geisler *et al.*, 2014).

In 1961, the very interesting observation that root geotropism is dependent on light in some plant species was first reported (Lake and Slack. 1961). Further studies on maize (Scott and Wilkins, 1969), *Vanilla planifolia* and two other vanilla species (Irvine and Freyre, 1961), and *Convolvulus arvensis* (Tepfer and Bonnett, 1972) confirmed the light dependency of root gravitropism. The importance of the root cap in sensing white light was subsequently investigated in maize (Wilkins and Wain, 1975). One or more phytochromes were suggested to be involved in this light-dependent gravitropism, because red light was most effective in inducing the gravitropic response (Tepfer and Bonnett, 1972; Shen-Miller, 1978). Moreover, analyses of the action spectrum of the light-induced gravitropic response in maize roots also suggested that a red light photoreceptor was involved (Suzuki and Fujii, 1978; Suzuki *et al.*, 1980). However, the gravitropism of maize roots did not exhibit red–far-red reversibility, suggesting that a non-phytochrome photoreceptor that perceived light at 640nm was involved. While, Miyazaki and colleagues determined that phytochrome perceived light at 630nm in the same maize cultivar (Miyazaki *et al.*, 1984). In contrast, other studies on different maize cultivars (e.g. *Z. mays* cv. Bear×W38 or cv. Merit) indicated that the red light-induced change in root gravitropism was due to a phytochrome exhibiting a very low fluence response, and was only partially reversible by far-red light (Mandoli *et al.*, 1984; Feldman and Briggs, 1987). The reason roots require light irradiation to exhibit gravitropism has not been fully characterized.

More recently, the constant touch-down and -up behaviours (i.e. crawling) of maize roots were documented in time-lapse analyses (Hahn *et al.*, 2006; Burbach *et al.*, 2012). The touch-down phase was enhanced by exposure to white light, and, when the root was inserted into a glass capillary column and inverted, white light exposure resulted in a ‘U-turn’ of the root inside the thin capillary tube (Burbach *et al.*, 2012). The U-turn of maize roots required thinning of the root apex. Analyses of the maize root U-turn behaviour suggested that this phenomenon is related to thigmotropism and gravitropism, and requires light and an intact root cap.

We previously determined that IAA was synthesized from tryptophan in the maize coleoptile tip (~0–3mm tip region) via the YUCCA (YUC) pathway, and that IAA was subsequently transported downward (Nishimura *et al.*, 2009, 2014). However, little is known about the mechanism controlling IAA levels in maize roots. Some studies have indicated that at least some of the IAA in the root apex is derived from the upper region of roots (Berleth and Sachs, 2001; Peer *et al.*, 2014). However, recent studies on Arabidopsis have indicated that IAA is also synthesized in root apices (Ljung *et al.*, 2005; Stepanova *et al.*, 2008; Chen *et al.*, 2014; Yang *et al.*, 2014).

In this study, analyses of light- and root cap-dependent root gravitropism revealed that light induced the accumulation of IAA in the transition zone of the maize root apex. This light-induced increase in IAA levels was necessary for roots to exhibit the U-turn behaviour. The increase in IAA abundance was strongly inhibited by co-treatment with two IAA biosynthesis inhibitors, namely l-kynurenine (Kyn) and yucasin. Treatments with these inhibitors enhanced root growth, but inhibited the U-turn. A tracer experiment with heavy isotope-labelled tryptophan suggested that some of the IAA in the root apex was synthesized *de novo* from tryptophan. Analyses of the transcript levels of several IAA biosynthesis genes, including tryptophan aminotransferase-related genes (e.g. *Zmtat*) and *YUCCA* genes (e.g. *Zmyuc*), in the maize root tip region indicated that genes encoding enzymes in the YUC pathway were involved in IAA synthesis in the root apex. Light-induced increases in IAA levels were observed to be essential for the light-dependent U-turn behaviour of maize roots.

## Materials and methods

### Plant materials and growth conditions

Maize seeds (*Zea mays* L. cv. Golden Cross Bantam 70 or B73) were sterilized in 0.5% (v/v) sodium hypochlorite, and then rinsed in running tap water for 18–20h. After imbibition, seeds were sown on moistened filter paper (3MM CHR, Whatman) and allowed to germinate for 2–2.5 d at 25 °C in darkness (Burbach *et al*., 2012). All experimental procedures were performed under dim green light (Mori *et al.*, 2005). Seeds of the *vt2* mutant were obtained from the Maize Genetics Cooperation Stock Center (http://www.uiuc.edu/ph/www/maize).

### Analyses of gravitropic curvature and root U-turn behaviour

To investigate the effects of light on the gravitropism of maize roots, dark-grown seedlings were placed horizontally on a wet floral sponge block (Water Absorption Sponge for Horticulture; DAISO, Hiroshima, Japan), and the gravitropic curvature of roots and coleoptiles was observed after a 20h incubation in darkness or under light (~36 µmol m^−2^ s^−1^). For the U-turn experiments, roots were inserted into glass capillary tubes (IK-PAS-5P, 5mm inner diameter; Iwaki, Shizuoka, Japan), which were then inverted (Burbach *et al.*, 2012). Seedlings were incubated in darkness or under light (~36 µmol m^−2^ s^−1^) for 1, 3, 6, 9, 12, or 20h. Root caps were removed using a blade, and the gravitropic responses of de-capped roots with or without light stimulation were observed. To ensure plant materials did not dry, all experiments were conducted in a box with ~5mm of water at the bottom. Additionally, high-humidity conditions were maintained by covering the box with plastic wrap.

For inhibitor experiments, whole roots of dark-grown seedlings were immersed in mock or inhibitor solutions for 60min. The roots were then inserted into glass capillary tubes, which were then inverted. After 18h, U-turns were observed and photographed. The mock solution was 10mM KPB (pH 6.8) containing 0.2% DMSO. The following inhibitors were prepared in KPB solution: 10 µM Kyn and 50 µM yucasin [IAA biosynthesis inhibitors (He *et al.*, 2011; Nishimura *et al.*, 2014)], 50 µM brefeldin A (BFA; intracellular trafficking inhibitor), 100 µM naphthylphthalamic acid (NPA; IAA efflux inhibitor), and 50 µM 1-naphthoxyacetic acid (1-NOA) and 50 µM ethyl 2-[(2-chloro-4-nitrophenyl) thio]acetate (7-B3) (Suzuki *et al.*, 2014) (IAA influx inhibitors).

### Time-lapse imaging of root U-turn behaviour

Maize roots were treated with mock solution or 50 µM yucasin+10 µM Kyn for 1h before being positioned in glass capillaries as described above. Roots were then incubated in an inverted orientation and photographed under either dark or light conditions. To obtain images in the dark, very dim green light was used as a safe light. Time-lapse photographs were taken at 5min intervals with a single-lens reflex camera (EOS Kiss x7i, Canon) with a programmable shutter controller (model E6315, Etsumi) during a 24h period. The pictures were converted into a sequential movie using the Graphic Converter 9 Lemke Software & QuickTime 7 Pro (Apple).

### Calculation of maize root growth rate

The position of the tip of each inverted maize root was marked on the glass capillary tube before incubation. After incubation for 18h in darkness or under light, the roots were photographed and root lengths before (length B) and after incubation (length A) were measured using Lenaraf220b.xls (Vector Japan, Tokyo, Japan). The growth rate (%) was calculated as follows: length (A−B)/(length B)×100.

### Quantification of IAA

To obtain root sections for IAA quantifications, roots (intact or de-capped; mock- or inhibitor-treated for 1h in darkness) were placed between two layers of filter paper (moistened with water, mock KPB, or inhibitor solutions), wrapped in plastic film, and incubated under the normal direction of gravity (i.e. not inverse) in darkness or under white light (~36 µmol m^−2^ s^−1^) for 1.5h. The root tips were then cut into 0–1, 0–3, or 0–5mm segments, which were immediately frozen in liquid N_2_ and stored at −80 °C. Endogenous IAA was extracted from the segments and quantified by GC-MS in selected ion monitoring mode (GC-SIM-MS) as described by Nishimura *et al.* (2014) with minor modifications. Briefly, the pH of the supernatants from crude extracts was adjusted to 3.0 with 0.1N HCl. Free IAA was then partitioned twice against ether. The ether phase was dried under a stream of N_2_ gas and the residue was dissolved in methanol. The IAA in the methanol solution was detected by HPLC and GC-SIM-MS. [^13^C_6_]IAA was used as an internal standard as previously described (Mori *et al.*, 2005). Free IAA was detected in 0–1mm tips (*n*=20–31), 0–3mm tips (*n*=6–10), and 0–5mm tips (*n*=4–6). The effect of the root cap on IAA levels in roots was assessed in the presence or absence of white light irradiation using intact or de-capped roots (five or six 0–3mm sections for each experiment). The IAA concentrations in the root tips were evaluated using four to six 0–5mm root tips after treatment with the following inhibitors: 50 µM yucasin+10 µM Kyn, 50 µM BFA, 100 µM NPA, and 50 µM 1-NOA+50 µM 7-B3.

### Incorporation of a [^13^C_11_^15^N_2_]tryptophan stable isotope into IAA in the root tip

The 0–5mm root tip region of 2.5-day-old etiolated maize seedlings was immersed in the following solutions for 30 min: 10mM KPB (pH 6.8) (mock), 50 µM yucasin+10 µM Kyn, 1mM [^13^C_11_^15^N_2_]tryptophan, and 50 µM yucasin+10 µM Kyn+1mM [^13^C_11_^15^N_2_]tryptophan. All of these reagents were prepared in 10mM KPB (pH 6.8). After incubation, the 0–10mm segments of root tips were collected. The endogenous IAA and ^13^C_9_^15^N_1_-labelled IAA contents were determined by GC-SIM-MS using [^13^C_6_]IAA as an internal standard as previously described (Nishimura *et al.*, 2009).

### Reverse transcription (RT)–PCR and quantitative RT–PCR (qPCR)

Total RNA was extracted from frozen tissues using an RNeasy Plant Mini Kit (Qiagen, Hilden, Germany). Contaminating DNA was removed using RNase-free DNase (Qiagen). The RNA (0.5 μg) was reverse transcribed using a High Capacity RNA-to-cDNA kit (Applied Biosystems, Foster City, CA, USA) according to the manufacturer’s protocol. The sequences of four *Zmtat* and 12 *Zmyuc* genes were obtained from the MaizeGDB database (http://www.maizegdb.org/) (Supplementary Table S1 at *JXB* online). Primers specific for the *Zmtat*, *Zmyuc*, and *Zmubiquitin* genes (Supplementary Table S2; Nishimura *et al.*, 2014) were used to amplify gene transcripts. The KOD FX DNA polymerase (Toyobo, Osaka, Japan) was used for the RT–PCR. The resulting amplified products were analysed by 3% agarose gel electrophoresis and visualized by ethidium bromide staining. qPCR was completed using a LightCycler 480 system (Roche, Basel, Switzerland) with the SYBR Green I Master Mix (Applied Biosystems). *Zmubiquitin* was used as the reference gene to normalize transcription levels. All qPCR experiments were performed using three biological replicates and two technical replicates. Melting and standard curves were prepared for each qPCR experiment.

## Results

### Light-dependent gravitropic curvature and the necessity of the root cap

**Fig. 1.**
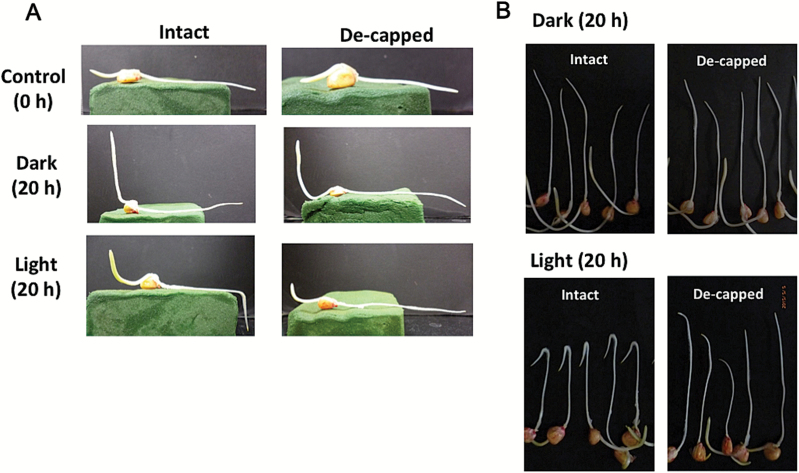
Light-dependent gravitropic response of maize roots and the necessity of the root cap. (A) Etiolated maize seedlings (2 d old) were placed horizontally on a wet floral foam block, and incubated for 20h in darkness or under white light. Left, with intact roots; right, with de-capped roots. (B) Roots of 2-day-old maize seedlings were inserted into capillary tubes and inverted, then kept for 20h in darkness or under white light. Roots were photographed after they were removed from capillary tubes. Top, 20h incubation in the dark; bottom, 20h incubation under white light. Left, intact roots; right, de-capped roots. (This figure is available in colour at *JXB* online.)

**Fig. 2.**
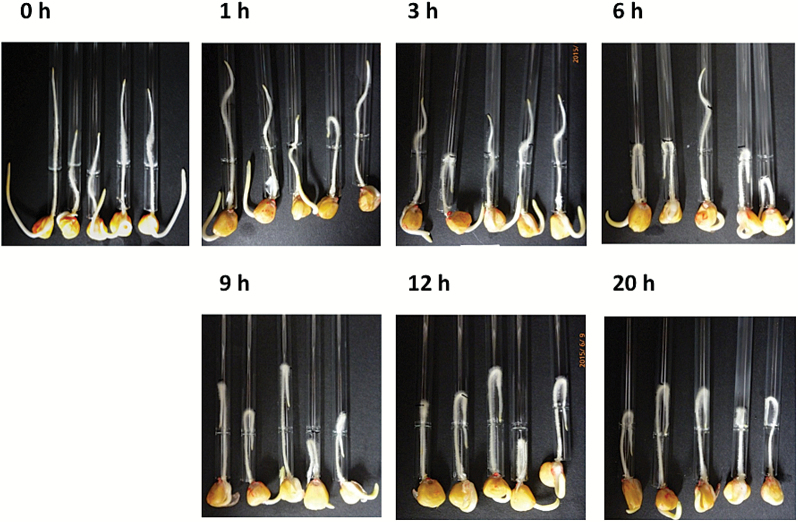
Effects of the duration of white light exposure on root U-turn behaviour. Roots of 2-day-old maize seedlings were inserted into capillary tubes, inverted under dim green light, exposed to white light for specific periods, and then incubated in darkness. (This figure is available in colour at *JXB* online.)

Etiolated maize seedlings (2 d old) were placed horizontally on a wet floral foam block, and then incubated for 20h in darkness or under white light (~36 µmol m^−2^ s^−1^). Although previous studies indicated that maize roots exhibited light-dependent gravitropic curvature after short-term irradiation (Shen-Miller, 1978; Mandori *et al.*, 1984), our results suggested that both light irradiation and the root cap were necessary for root gravitropism (i.e. an almost 90 ° curvature) under continuous light irradiation (Fig. 1A). In contrast, the aerial parts (i.e. coleoptile) exhibited clear curvature against gravity both in darkness and under light (Fig. 1A). When roots in capillary tubes were inverted and incubated for 20h in darkness or under white light, only intact roots under white light exhibited U-turn behaviour (Fig. 1B). Because the root was inverted within the capillary tube, the U-turn behaviour required both gravitropic and thigmotropic responses. In fact, continuous exposure to light for >6h was required for complete U-turns to occur (Fig. 2).

### Light irradiation increased IAA levels in root tips

**Fig. 3.**
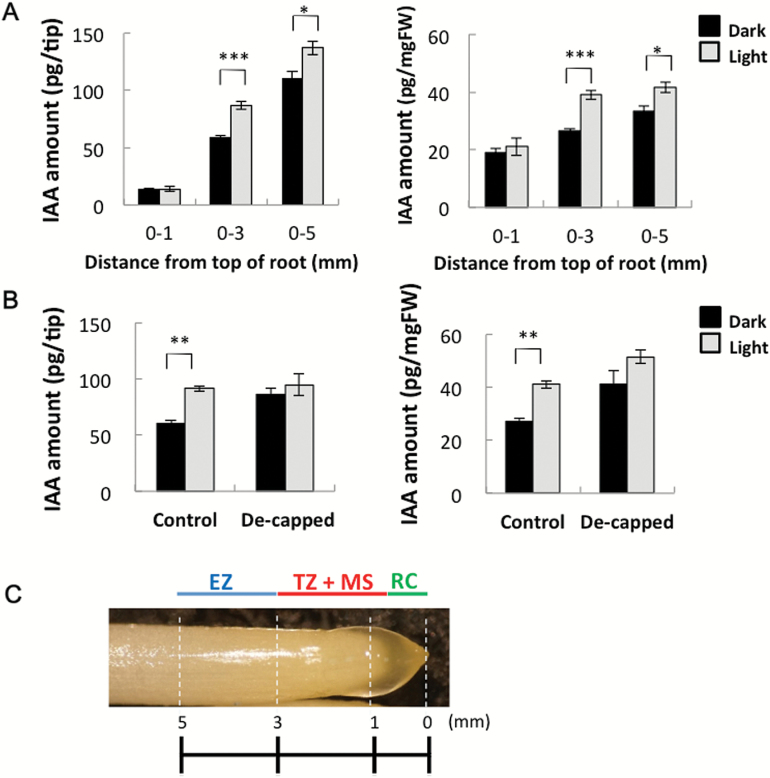
Light irradiation increased IAA levels in root tips. Etiolated maize seedlings (2 d old) were incubated under white light (for 1.5h; white column) or in darkness (black column). (A) Abundance of IAA in 0–1, 0–3, and 0–5mm maize root tip regions [per tip (left) and per mg fresh weight (right)]. Values are presented as the mean ±SE (0–1mm, *n*=3; 0–3mm, *n*=8; 0–5mm, *n*=3). Asterisks indicate significant differences between roots treated in darkness and light-irradiated roots (Student’s *t*-test; ***P<0.001; *P<0.05). (B) Effect of de-capitation on IAA levels in the 0–3mm root tip region. Data were obtained from two independent experiments. Values are presented as the mean ±SE (4≤*n*≤5). Asterisks indicate significant differences between roots treated in darkness and light-irradiated roots (**P< 0.01). (C) Photo of the maize root apex and schematic views of root structure. EZ, elongation zone; TZ, transition zone; MS, meristem; RC, root cap. (This figure is available in colour at *JXB* online.)

Etiolated maize seedlings (2 d old) were incubated under white light or in darkness for 1.5h, and then IAA contents in the 0–1, 0–3, and 0–5mm tip regions were determined (Fig. 3A). The 0–1mm region mainly consisted of the root cap and meristem region, the 1–3mm region mainly consisted of the meristem and the transition zone, and the 3–5mm region mainly consisted of the elongation zone (Fig. 3C). In dark-grown roots, the abundance of IAA per tip increased from 13 pg to 110 pg along the root length, while the IAA content per mg fresh weight exhibited a much smaller increase (i.e. from ~20 pg to 30 pg; Fig. 3A). This result indicated that IAA was distributed relatively evenly throughout the 0–5mm region. Light irradiation did not affect IAA abundance in the 0–1mm region, while the IAA contents in the 0–3mm and 0–5mm regions were significantly higher in light-irradiated roots than in dark-incubated roots (Student’s *t*-test; *P*=0.000005 and *P*=0.03, respectively). The increase in IAA abundance in the 0–3mm segment (i.e. ~24 pg per tip) was almost the same as that in the 0–5mm segment (i.e. 26 pg per tip). This strongly suggested that the light-induced IAA increase was restricted to the 1–3mm region. The effect of removing the root cap on the IAA level in the 0–3mm region with or without light irradiation was determined (Fig. 3B). Decapitation resulted in increased IAA levels in non-irradiated roots, but not in irradiated roots, suggesting that the root cap was essential for the light-dependent IAA increase in the 0–3mm root apex region.

### Yucasin and Kyn decreased IAA levels in maize roots

**Fig. 4.**
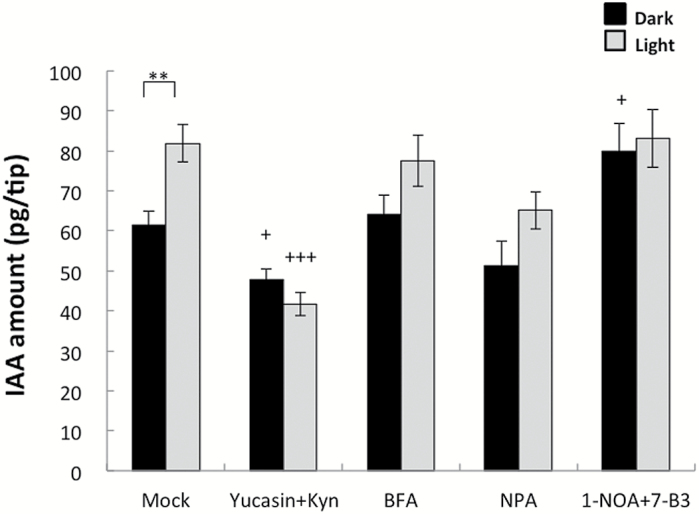
Effects of several IAA-related inhibitors on IAA levels in the 0–3mm root apex region. Whole roots of 2-day-old etiolated maize seedlings were immersed in mock [10mM KPB (pH 6.8)] or inhibitor solutions for 1h. The following inhibitors were prepared in 10mM KBP: 50 µM yucasin+10 µM Kyn, 50 µM BFA, 100 µM NPA, and 50 µM 1-NOA+50 µM 7B-3. Roots were then placed on wet filter paper moistened with inhibitor solution, wrapped in plastic film, and incubated in darkness or under white light for 1.5h. Root tips (0–3mm, *n*=4–6) were collected for each treatment to quantify IAA. Data were obtained for at least two independent experiments. Values are presented as the mean ±SE (5≤*n*≤10). Asterisks indicate significant differences between roots treated in darkness and light-irradiated roots (Student’s *t*-test; **P<0.01). Plus symbols indicate significant differences between the mock control and inhibitor treatments (+*P*<0.05; +++*P*<0.001).

To determine how IAA abundance is affected by light, the effects of several IAA-related inhibitors on IAA levels in the 0–3mm region of the root apex were investigated (Fig. 4). The inhibitors included two IAA biosynthesis inhibitors (i.e. yucasin and Kyn), an intracellular trafficking inhibitor (i.e. BFA), and IAA transport inhibitors (i.e. NPA, 1-NOA, and 7-B3). In previous studies of Arabidopsis, co-treatments with yucasin and Kyn at relatively low concentrations inhibited the indole-3-pyruvic acid pathway without causing any toxic effects (Hayashi *et al.*, 2014; Nishimura *et al.*, 2014). In the current study, co-treatment with yucasin and Kyn decreased IAA levels by ~23% in non-irradiated roots and by 55% in light-irradiated roots. No drastic changes in IAA levels were observed in roots treated with the other inhibitors, but NPA slightly reduced IAA levels under light conditions and 1-NOA+7-B3 increased IAA levels in darkness. These responses differed markedly from those observed in coleoptile tips, in which NPA increased IAA levels, and treatment with yucasin decreased IAA abundance by ~20% after a 1h incubation (Nishimura *et al.*, 2009, 2014). These data indicate that coleoptiles and roots harboured partly similar IAA biosynthesis mechanisms, but had different mechanisms regulating IAA movement.

### Incorporation of a stable isotope label into IAA after uptake of [^13^C_11_^15^N_2_]tryptophan in the root tip

**Fig. 5.**
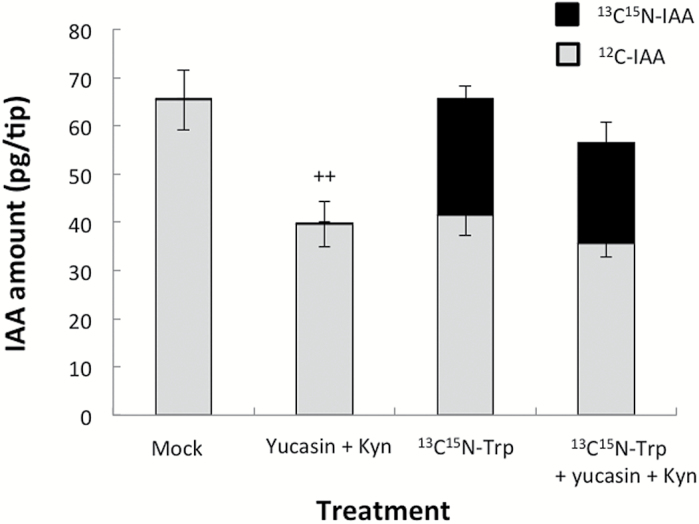
Incorporation of a stable isotope label into IAA after uptake of [^13^C_11_^15^N_2_]tryptophan in the root tip. Maize root tips (0–5mm region) were immersed in mock (10mM KPB) or inhibitor solutions for 30min. The following inhibitors were prepared in KPB: 50 µM yucasin+10 µM Kyn, 1mM [^13^C_11_^15^N_2_]tryptophan, and 50 µM yucasin+10 µM Kyn+1mM [^13^C_11_^15^N_2_]tryptophan. Root tips (0–10mm region) were then collected (two per treatment) to quantify IAA. Grey bar, endogenous IAA; black bar, ^13^C_9_^15^N_1_-labelled IAA. Values are presented as the mean ±SE (*n*=5 or 6). Plus symbols indicate significant differences between the mock control and yucasin+Kyn treatments (Student’s *t*-test; ++*P*<0.01).

We previously determined that in maize coleoptile tips, IAA was actively synthesized *de novo* from exogenously supplied [^13^C_11_^15^N_2_]tryptophan. This indicated that IAA was vigorously synthesized in the coleoptile tip from tryptophan via the YUC pathway (Nishimura *et al.*, 2009). In the present study, co-treatment with yucasin and Kyn significantly decreased IAA abundance in the root apex (Fig. 4). Therefore, incorporation of an isotope-labelled tryptophan into IAA in the maize root apex was monitored. When etiolated maize roots were treated with labelled tryptophan ([^13^C_11_^15^N_2_]tryptophan), the labelled IAA accounted for ~30% of the total IAA content after a 30min incubation. However, the overall IAA level was the same as that in the mock control (i.e. ~65 pg per tip) (Fig. 5). Co-treatment with yucasin and Kyn decreased the amount of IAA in roots by ~40% after a 30min incubation. The reduction rate was similar to that observed in a previous study when maize coleoptile tips were treated with 50 µM yucasin for 30min (Nishimura *et al.*, 2014). These results suggested that at least some of the IAA in roots is synthesized from tryptophan via the YUC pathway. However, co-treatments of yucasin+Kyn with 1mM ^1^[^13^C_11_^15^N_2_]tryptophan resulted in only small decreases in total IAA abundance and rate of incorporation. The reason for this is that Kyn is no longer able to work in the presence of high doses of tryptophan, as mentioned by He *et al.* (2011).

### Expression of IAA biosynthesis genes in maize root tip regions

**Fig. 6.**
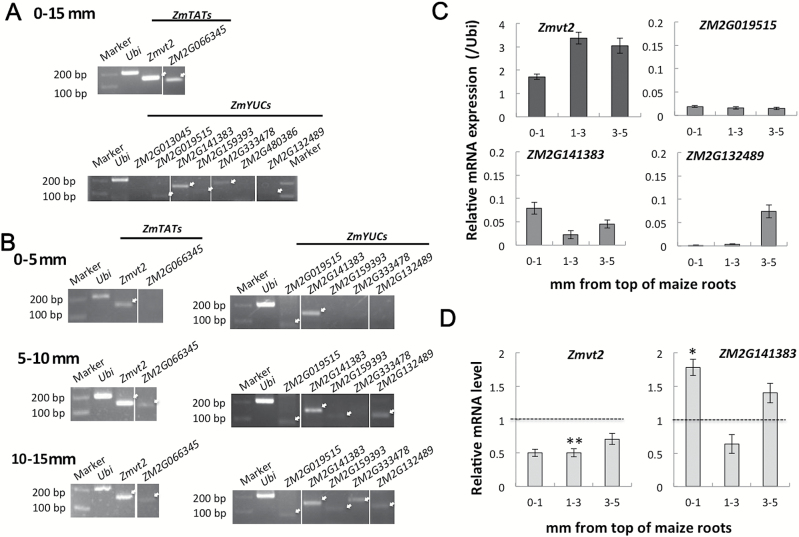
Transcript profiles of *Zmtat* and *Zmyuc* genes in maize roots. (A) Transcription levels of *Zmtat* and *Zmyuc* genes in the 0–15mm root tip region. Approximately 200ng of total RNA was used for the RT–PCR. *Ubi*, *Zmubiquitin*. (B) Transcription of two *Zmtat* and five *Zmyuc* genes was analysed by RT–PCR. Total RNA was obtained from the 0–5, 5–10, and 10–15mm root regions. Approximately 50ng of total RNA was used for the RT–PCR. (C) Total RNA was extracted from the 0–1, 1–3, and 3–5mm root tip regions and used for qPCR. *Zmubiquitin* served as the internal standard. Values are presented as the mean ±SE (3≤*n*≤5). (D) Effects of light irradiation on the transcription of *Zmvt2* and *Zmyuc* genes. *Zmubiquitin* served as the internal standard for qPCR. Transcript levels of samples treated in darkness were set to 1 and the relative transcript levels for samples exposed to light are presented. Asterisks indicate significant differences between roots treated in darkness and light-irradiated roots (Student’s *t*-test; *P<0.05; **P<0.01).

To confirm further the involvement of the YUC pathway in IAA biosynthesis in maize roots, the expression profiles of the *Zmtat* and *Zmyuc* genes were investigated (Fig. 6; Supplementary Table S1). We first checked their expression levels using the Maize eFP Browser (Winter *et al.*, 2007; Sekhon *et al.*, 2011). We identified nine putative root-expressed genes, comprising two *Zmtat* and seven *Zmyuc* genes (Supplementary Fig. S1). Their transcript levels within 15mm of the primary root tips were determined by RT–PCR (Fig. 6A). Transcripts for two *Zmtat* genes (i.e. *Zmvt2* and *ZM2G066345*) and five *Zmyuc* genes (i.e. *ZM2G019515*, *ZM2G141383*, *ZM2G159393*, *ZM2G333478*, and *ZM2G132489*) were detected in this region (Fig. 6A; white arrows). Expression of these genes was further analysed in 0–5, 5–10, and 10–15mm root tip regions (Fig. 6B). In the 0–5mm segment, transcripts for *Zmvt2*, *ZM2G019515*, and *ZM2G141383* were detected. In the 5–10mm region, *ZM2G066345*, *ZM2G159393*, and *ZM2G132489* transcripts were observed. In the 10–15mm region, transcripts for the two *Zmtat* and five *Zmyuc* genes were detected.

We completed qPCR analyses to monitor the transcription profiles of *Zmvt2* and three *Zmyuc* genes (i.e. *ZM2G019515*, *ZM2G141383*, and *ZM2G132489*) in the tip, as well as the 0–1, 1–3, and 3–5mm regions (Fig. 6C). The *Zmvt2* transcript level was higher in the 1–5mm segment than in the 0–1mm tip region. Thus, it is possible that the *Zmvt2* gene product influences the increase in IAA abundance in the transition zone after light irradiation. Additionally, among the *Zmyuc* genes, *ZM2G141383* was the most highly transcribed, with the greatest abundance observed in the 0–1mm region, which includes the root cap (Fig. 6C). Very low *ZM2G019515* transcript levels were observed throughout the root tip regions, while the *ZM2G132489* transcript was detected in the 3–5mm region, but not in the 0–1mm or 1–3mm regions.

The fact that light irradiation increased IAA levels in the 1–3mm root region (Fig. 3A) suggests that light may induce the transcription of the IAA biosynthetic genes *Zmvt2* and *ZM2G141383*, which are expressed in this region of the root apex. Therefore, we further examined the effect of light irradiation on the transcription of these genes in the root tip region (Fig. 6D), and determined that light decreased *Zmvt2* transcript levels. In contrast, light irradiation increased the transcription of *ZM2G141383*, especially in the 0–1mm region. Consistent with these findings, we observed light-dependent root gravitropism and U-turn behaviour in *vt2* mutant roots. However, the gravitropic response of mutant roots was the same as that of the control maize roots (Supplementary Fig. S2). Although these results suggest that ZmVT2 has no critical role in light-induced increases in IAA levels, it is possible that the higher *ZM2G141383* transcription level stimulated by light influences the increase in IAA abundance in the 0–1mm root tip region. This implies that IAA biosynthesis occurs in the 0–1mm tip region, and that IAA moves from the root tip to the transition zone.

### Effects of IAA biosynthesis inhibitors on light- and root cap-dependent U-turn behaviour of maize root apices

**Fig. 7.**
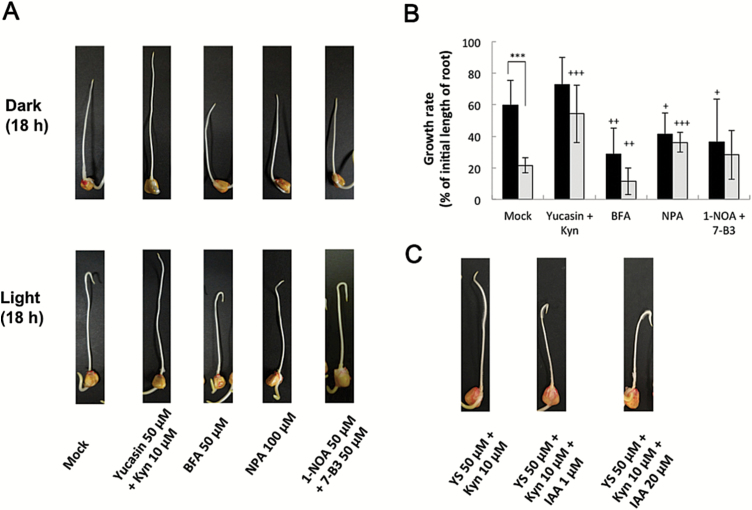
Effects of several IAA-related inhibitors on root U-turn behaviour and growth. (A) Whole roots of 2-day-old maize seedlings were pre-treated with inhibitors for 1h as described in the legend of Fig. 4. Roots were then inserted into capillary tubes, inverted, and incubated for 18h in darkness or under white light. For each treatment, at least three independent experiments with 4–6 seedlings were completed. Images and movies of U-turn behaviour are provided in the Supplementary data. (B) Growth rates of maize roots presented in (A). Data were obtained from two or three independent experiments (each with 9–11 roots). Black column, in darkness; white column, under white light. Values are presented as the mean ±SE. Asterisks indicate significant differences between roots treated in darkness and under light (Student’s *t*-test; *P*=0.00006). Plus symbols indicate significant differences between the mock control and chemical treatments (Student’s *t*-test; +*P*<0.05; ++*P*<0.01; +++*P*<0.001). (C) Effects of co-treatment with IAA and yucasin+Kyn on root U-turn behaviour. Samples were treated as described in (A). (This figure is available in colour at *JXB* online.)

The effects of various inhibitors on light- and root cap-dependent U-turn behaviour of maize roots were investigated (Fig. 7A; Supplementary Movie S1; Supplementary Fig. S3). When the IAA biosynthesis inhibitors yucasin or Kyn were applied individually, they weakly affected the U-turn behaviour of roots (data not shown). However, co-treatment with yucasin and Kyn strongly inhibited the U-turn behaviour and significantly stimulated root elongation. The IAA efflux inhibitor NPA inhibited root U-turns, but BFA, an inhibitor of vesicular trafficking, did not. The IAA influx inhibitors 1-NOA and 7-B3 did not significantly inhibit root U-turns (Fig. 7A; Supplementary Fig. S3). The effects of these inhibitors on the root growth rate were also investigated (Fig. 7B). Light irradiation inhibited root elongation in the mock control (Student’s *t*-test; *P*=0.00006). Additionally, a co-treatment with yucasin and Kyn in darkness only slightly stimulated root elongation. In contrast, this co-treatment strongly stimulated root elongation under light conditions relative to the root elongation rate observed for the mock control (Student’s *t*-test; *P*=0.0002). This result indicated that inhibited IAA biosynthesis decreased the IAA levels in the tip region, which promoted root elongation and strongly inhibited the U-turn behaviour of roots without causing toxic effects on root growth (Fig. 7; Supplementary Movie S1). Treatment with BFA inhibited root elongation to about half of that observed in the mock control both in darkness and under light (Student’s *t*-test; *P*=0.0012 and *P*=0.0099, respectively). Exposure to NPA also inhibited root elongation in darkness, but promoted it under light (Student’s *t*-test; *P*=0.0182 and *P*=0.00003, respectively). The effects of 1-NOA+7-B3 on root growth were similar to those of NPA, although there were significant differences for treatments in darkness (Student’s *t*-test; *P*=0.0358). Additionally, we investigated the effects of exogenous IAA on the inhibition of root U-turns in seedlings treated with yucasin+Kyn (Fig. 7C). The application of 1 µM and 20 µM IAA clearly resulted in the recovery of the U-turn behaviour in seedlings treated with yucasin+Kyn. These findings suggest that IAA biosynthesis is critical and the IAA transport systems might have a role in the light-induced U-turn behaviour of maize roots.

## Discussion

### Light- and root cap-dependent gravitropic curvature and U-turn behaviour of maize root apices

It has long been known that light and the root cap are required for maize root gravitropism. The requirement of light for gravitropism has also been demonstrated for some other plant species (Irvine and Freyre, 1961; Tepfer and Bonnett, 1972). This is possibly an adaptive response of specific plants with aerial roots (Shen-Miller, 1978). However, it is also possible that the light fluence required to trigger the gravitropic response differs among plant species. Further studies using different plants will provide new insights regarding light-dependent gravitropism.

Several studies have revealed that a relatively long exposure (2–6h) to white light was required for the gravitropic curvature of maize roots to a maximum angle of ~60 ° (Wilkins and Wain, 1975; Pilet, 1979). However, those studies did not analyse root curvature over periods longer than 6h. In the present study, analyses of the time dependency of the U-turn indicated that white light irradiation for at least 3–6h, followed by incubation in darkness for up to 20h was required for a complete U-turn (Fig. 2). The inverted roots exhibited U-turn behaviour only after the curvature exceeded 90 ° under light conditions. According to Pilet (1979), the gravitropic curvature of maize required an ~90min pre-treatment with white light, and the ‘memory’ of the perception of curvature completely disappeared after exposure to darkness for 5h. In the present study, even after a 120min exposure to white light, the root curvature was only ~40–60 ° (Supplementary Fig. S4). After incubation under light conditions for 3h, the roots grew straight (Fig. 2). It is likely that the irradiation time was too short for the roots to exhibit a U-turn, and the following incubation in darkness cancelled the light-induced response. Therefore, it seems that a longer period of light irradiation is required for the completion of the U-turn. Additionally, because the U-turns occurred within narrow capillary tubes, negative thigmotropism also affected the root-bending behaviour (Darwin and Darwin, 1880; Burbach *et al.*, 2012).

In Arabidopsis, root cap columella cells are the site of gravity perception. The results of the present study with maize roots indicated that an intact root cap is necessary for both the U-turn and root gravitropism, which is consistent with the findings of a previous study on a different maize cultivar (Burbach *et al.*, 2012). It is possible that the de-capped roots failed to perceive gravity, and thus could not complete the U-turn. In addition to its role in sensing gravity and/or light, the maize root cap is required for the initial redistribution of IAA stimulated by gravity (Young *et al.*, 1990). The asymmetric movement of IAA from the columella cells to the lateral root cap is important for gravitropism in Arabidopsis (Ottenschläger *et al.*, 2003), and is mediated by the re-localization of PIN-FORMED3 and 7 (Friml *et al.*, 2002; Kleine-Vehn *et al.*, 2010). Additionally, AUX1 and PIN2 mediate IAA flow from the lateral root cap and epidermis to the elongation zone (Swarup *et al.*, 2005; Žádníková *et al.*, 2015). These observations suggest that the lack of a U-turn in de-capped roots is due not only to the inability to perceive gravity and/or light but also to defective IAA transport. The results of the current study indicate that BFA and the co-treatment with 1-NOA and 7-B3 have no significant effects on maize root U-turn behaviour, while NPA has an inhibitory effect. Because the movement of IAA in the root tip region is regulated by highly complex and well-organized transport systems, detailed cellular and subcellular analyses of transcellular IAA movements in the root apex zones are necessary.

### Biosynthesis of IAA in the root apex

Previous studies on Arabidopsis and other plants have revealed that IAA moves downward (i.e. toward the roots) and is then redirected upward (i.e. toward the shoots) at the root cap (Ohwaki and Tsurumi, 1976; Tsurumi and Ohwaki, 1978; Hasenstein and Evans, 1988; Petrášek and Friml, 2009; Peer *et al.*, 2014). However, IAA biosynthesis was also observed to occur in maize and Arabidopsis root tips (Feldman, 1980; Ljung *et al.*, 2005; Chen *et al.*, 2014). In this study, the possibility of *de novo* IAA biosynthesis in maize roots was investigated using various inhibitors. Quantification of IAA by GC-MS revealed that co-treatment with yucasin and Kyn decreased IAA levels by ~50% in the root tip regions under light conditions (Fig. 4). Furthermore, heavy isotope-labelled tryptophan was incorporated into IAA in the 0–5mm tip region within 30min, indicating that some *de novo* IAA synthesis occurred in this region (Fig. 5). In another study, [^14^C]tryptophan was incorporated into IAA in the 0–5mm region of isolated maize root tips, and root cap removal enhanced this process (Feldman, 1980). This is consistent with our results for intact roots (Fig. 3), where the 1–3mm region (i.e. the transition zone) was observed to be the site of increased IAA abundance after exposure to light.

The transcript levels of genes related to IAA biosynthesis were monitored in the root apex (Fig. 6). *Zmvt2* and three *Zmyuc* genes (*ZM2G141383*, *ZM2G132489*, and *ZM2G019515*) are transcribed in the root apex (0–5mm). *Zmvt2* encodes a grass-specific tryptophan aminotransferase that is highly similar to tryptophan aminotransferases encoded by *AtTAA1*, *AtTAR1*, and *AtTAR2* in Arabidopsis (Phillips *et al.*, 2011). In Arabidopsis, *AtTAA1* and *AtTAR2* were observed to be expressed in the root meristem region (Stepanova *et al.*, 2008). We detected the *Zmvt2* transcript in maize roots (Fig. 6A, B), with the highest levels observed in the 1–3mm transition zone (Fig. 6C). The *Zmyuc* genes *ZM2G141383*, *ZM2G132489*, and *ZM2G019515* are similar to *AtYUC8*, *AtYUC3*, and *AtYUC10*, respectively (Fig. 6; Supplementary Table S1; Supplementary Fig. S5). *AtYUC10* is expressed at low levels in roots (Cheng *et al.*, 2007). The expression of *AtYUC8* is restricted to the root tip region, while *AtYUC3* is expressed in the upper regions of the root (Chen *et al.*, 2014). These observations combined with the results regarding inhibited IAA accumulation by yucasin+Kyn co-treatment suggest that at least some IAA is constantly synthesized in roots via the YUC pathway, which is a highly conserved pathway among plant species.

### Light-dependent increases in IAA abundance in the root apex

When roots were exposed to light, the IAA content increased in the 1–3mm root apex region (Fig. 3), which consists of the transition zone and the apical meristem (Fig. 3C). Several recent studies highlighted the importance of the root apex, especially the transition zone, for root growth and behaviour (Masi *et al.*, 2009; Baluška and Mancuso, 2013; Kagenishi *et al.*, 2016). Other studies revealed the influence of light on roots (Yokawa *et al.*, 2013, 2014, 2016). We observed that co-treatment with yucasin and Kyn significantly decreased the IAA levels in the root apex, especially in light-irradiated roots (Fig. 4), which inhibited the U-turn behaviour of roots and enhanced root growth (Fig. 7). This suggests that exposure to light induces *de novo* IAA biosynthesis and accumulation of IAA in the root apex. Root elongation stimulated by Kyn was also observed for *Brachypodium distachyon*, which is another monocotyledonous plant (Pacheco-Villalobos *et al.*, 2013). Additionally, transcripts of genes related to IAA biosynthesis were detected in the root tip region (Fig. 6), indicating the presence of *de novo* IAA biosynthesis via the YUC pathway. The expression of *AtTIR2* and *AtTAA1* in Arabidopsis root is regulated by gravity and aluminium treatment, respectively, suggesting their importance for local IAA synthesis to decrease IAA concentration gradients in the root tip region (Yamada *et al.*, 2009; Yang *et al.*, 2014). Our results indicated that *Zmvt2* transcript levels decreased following light irradiation (Fig. 6D). Additionally, the roots of the *vt2* mutant exhibited light-dependent gravitropic curvature and U-turn behaviour (Supplementary Fig. S2). These data indicate that ZmVT2 may be unnecessary for root U-turn behaviour, and that other ZmTAT enzymes may have redundant functions [e.g. *ZM2G066345*, which is transcribed more in the 5–15mm region than in the 0–5mm root region (Fig. 6B)]. In contrast, the transcription of one *Zmyuc* gene, *ZM2G141383* (*AtYUC8-like*), was up-regulated by light irradiation, suggesting that the changes in *Zmyuc* expression due to light irradiation may cause light-induced IAA accumulation (Fig. 6D). Light-induced increased *ZM2G141383* transcription was detected in the 0–1mm root tip region, which indicated the importance of the root cap and apical meristem. It is possible that the IAA synthesized in the 0–1mm root tip region moves to the upper 1–3mm transition zone, and the resulting IAA gradient is responsible for the gravitropic curvature. Additionally, exogenous IAA treatment could recover the U-turn behaviour inhibited by yucasin and Kyn (Fig. 7C). This particular finding supports the importance of IAA biosynthesis in roots via the YUC pathway for light-dependent root gravitropism and root U-turn behaviour.

In conclusion, our results indicate that changes to the IAA content in the transition zone induced by exposure to light are necessary for the U-turn behaviour of the maize root apex. Because this behaviour relies on gravitropism and thigmotropism, additional studies are required to clarify the mechanisms regulating light-dependent gravitropism and light-induced IAA accumulation. The completion of spatiotemporal studies on the expression patterns of IAA biosynthesis genes and localization of IAA transport proteins will clarify how IAA gradients are established by light irradiation. Future studies should attempt to identify the light receptor at the root cap (Feldman and Briggs, 1987; Mo *et al.*, 2015) involved in the light-dependent gravitropism in maize root apices. Finally, as Arabidopsis roots also synthesize IAA in their root apices (Ljung *et al.*, 2005; Stepanova *et al.*, 2008; Chen *et al.*, 2014; Yang *et al.*, 2014), it will be important to test possible effects of the light-induced IAA on root gravitropism in Arabidopsis.

## Supplementary data

Supplementary data are available at *JXB* online.

Table S1. Sequence details for four *Zmtat* and 12 *Zmyuc* genes obtained from the MaizeGDB database.

Table S2. Primers used for RT–PCR and qPCR.

Movie S1. Time-lapse movie of U-turn behaviour in darkness and under light.

Figure S1. Tissue-specific transcription patterns of *Zmtat* and *Zmyuc* genes obtained from the Maize eFP Browser (http://bar.utoronto.ca/efp_maize/cgi-bin/efpWeb.cgi).

Figure S2. Gravitropic response and U-turn behaviour of the maize *vt2* mutant.

Figure S3 Photos of maize roots in several independent U-turn experiments.

Figure S4. Effects of shorter durations of white light exposure on the gravitropic curvature of maize roots.

Figure S5. Phylogenetic analysis of amino acid sequences of tryptophan aminotransferase-related and YUCCA proteins.

Supplementary Data
