# Supplementary material for: Root cap-dependent gravitropic U-turn of maize root requires light-induced auxin biosynthesis via the YUC pathway in the root apex
Source: J Exp Bot. 2016 Jun 15;67(15):4581–91. doi: 10.1093/jxb/erw232 (PMC4973731; doi:10.1093/jxb/erw232)
Supplement: Supplementary Data [file supp_67_15_4581__index.html]

Root cap-dependent gravitropic U-turn of maize root requires light-induced auxin biosynthesis via the YUC pathway in the root apex — Root cap-dependent gravitropic U-turn of maize root requires light-induced auxin biosynthesis via the YUC pathway in the root apex — Supplementary Data 

# Root cap-dependent gravitropic U-turn of maize root requires light-induced auxin biosynthesis via the YUC pathway in the root apex

## Supplementary Data

Data files

- supplementary\_figures\_S1\_S5\_Tables\_S1\_S2.pdf - Supplementary Data
- supplementary\_Movie\_S1.mov - Supplementary Data
- supplementary\_Movie\_S2.mov - Supplementary Data
