# Supplementary material for: Root cap-dependent gravitropic U-turn of maize root requires light-induced auxin biosynthesis via the YUC pathway in the root apex
Source: J Exp Bot. 2016 Jun 15;67(15):4581–91. doi: 10.1093/jxb/erw232 (PMC4973731; doi:10.1093/jxb/erw232)
Supplement: Supplementary Data [file supp_erw232_supplementary_figures_S1_S5_Tables_S1_S2.pdf]

SUPPLEMENTARY FIGURES & TABLES

Figure S1 (1).

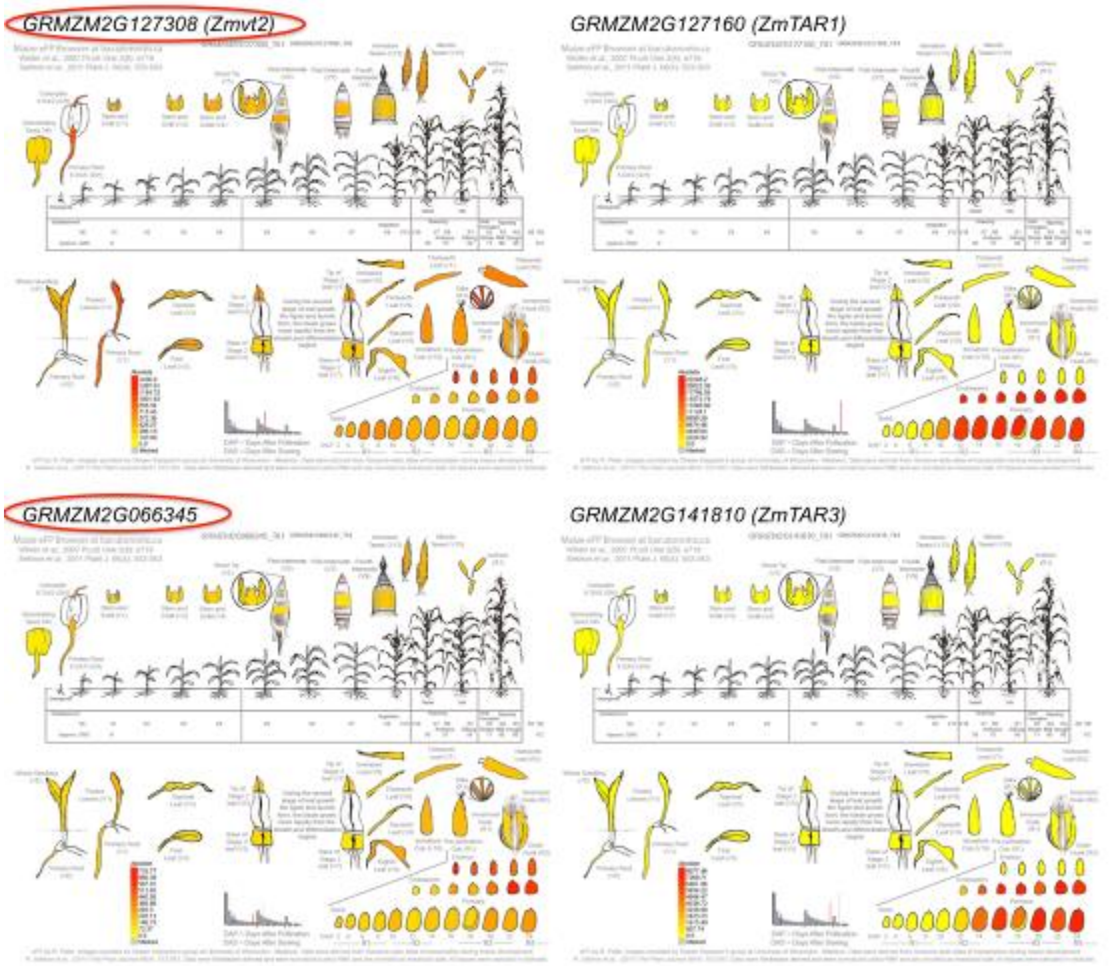

Figure S1 (2).

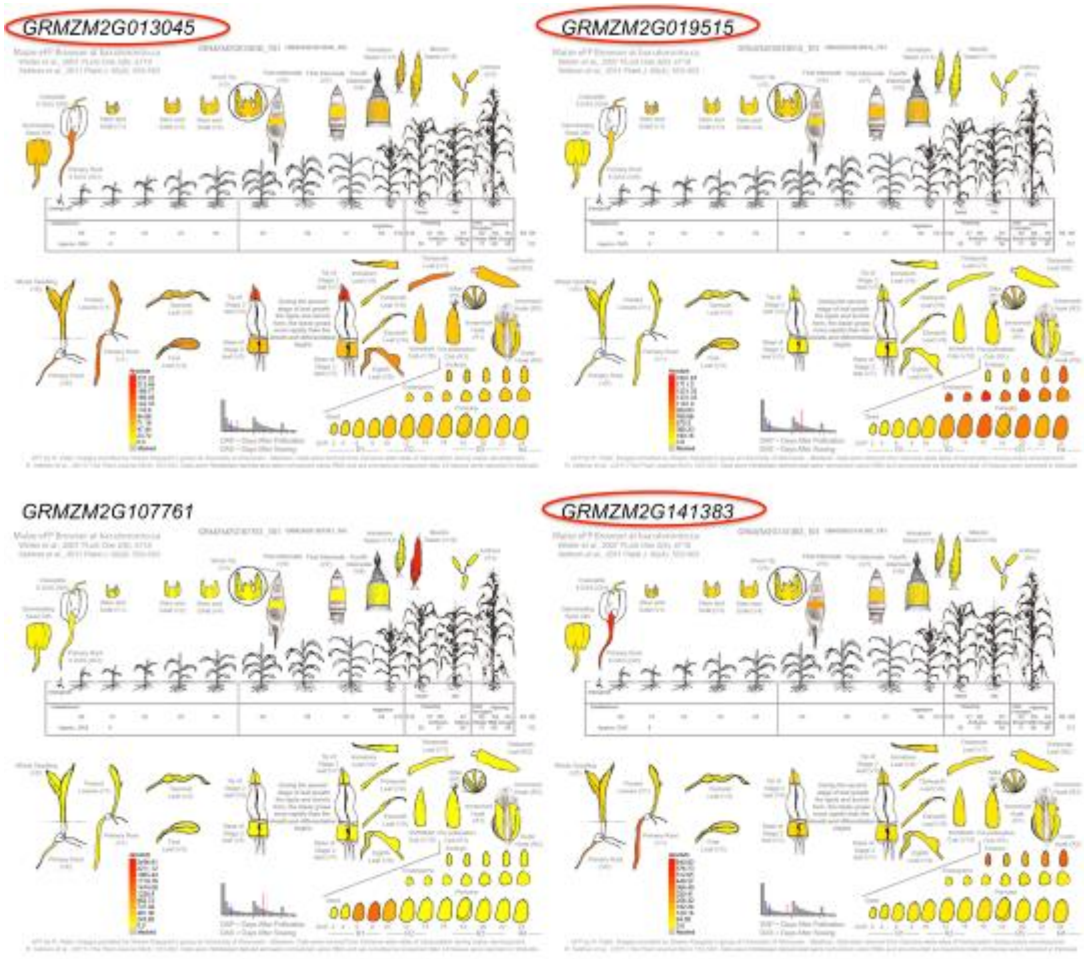
GRMZM2G013045
GRMZM2G019515
GRMZM2G107761
GRMZM2G141383

Figure S1 (3).

GRMZM2G159393

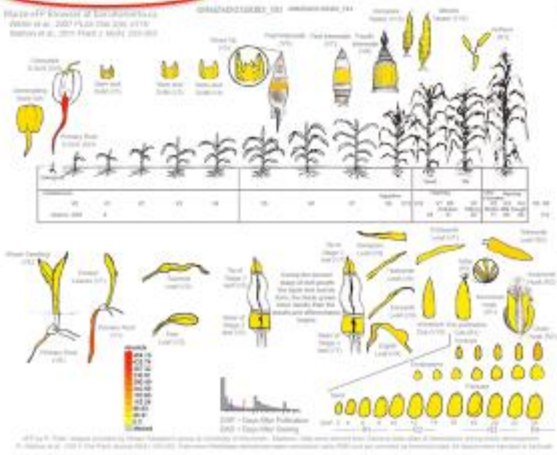

GRMZM2G328780

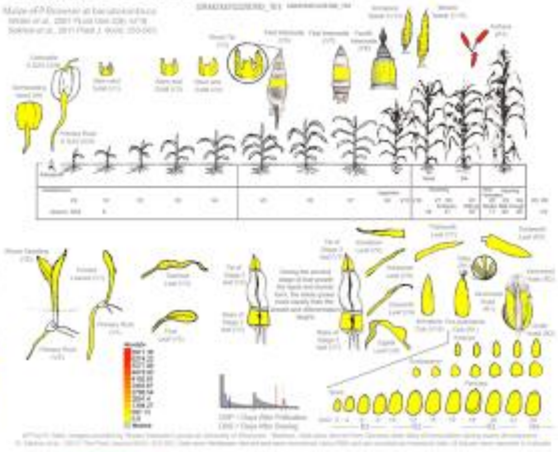

GRMZM2G333478

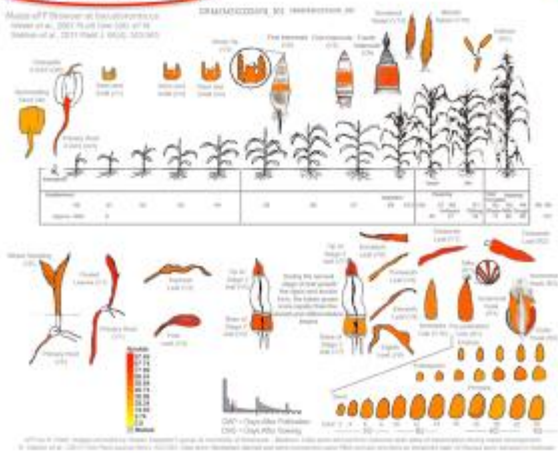

GRMZM2G480386

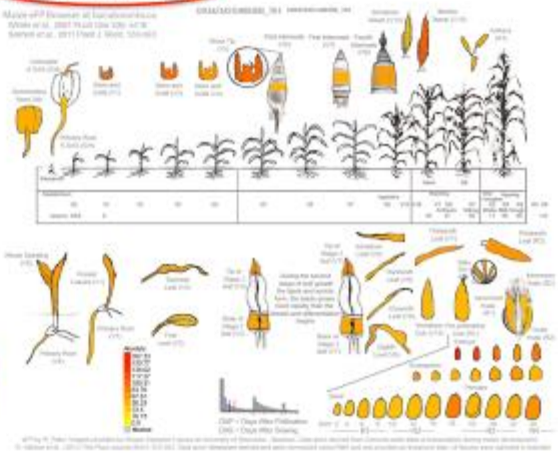

Figure S1 (4).

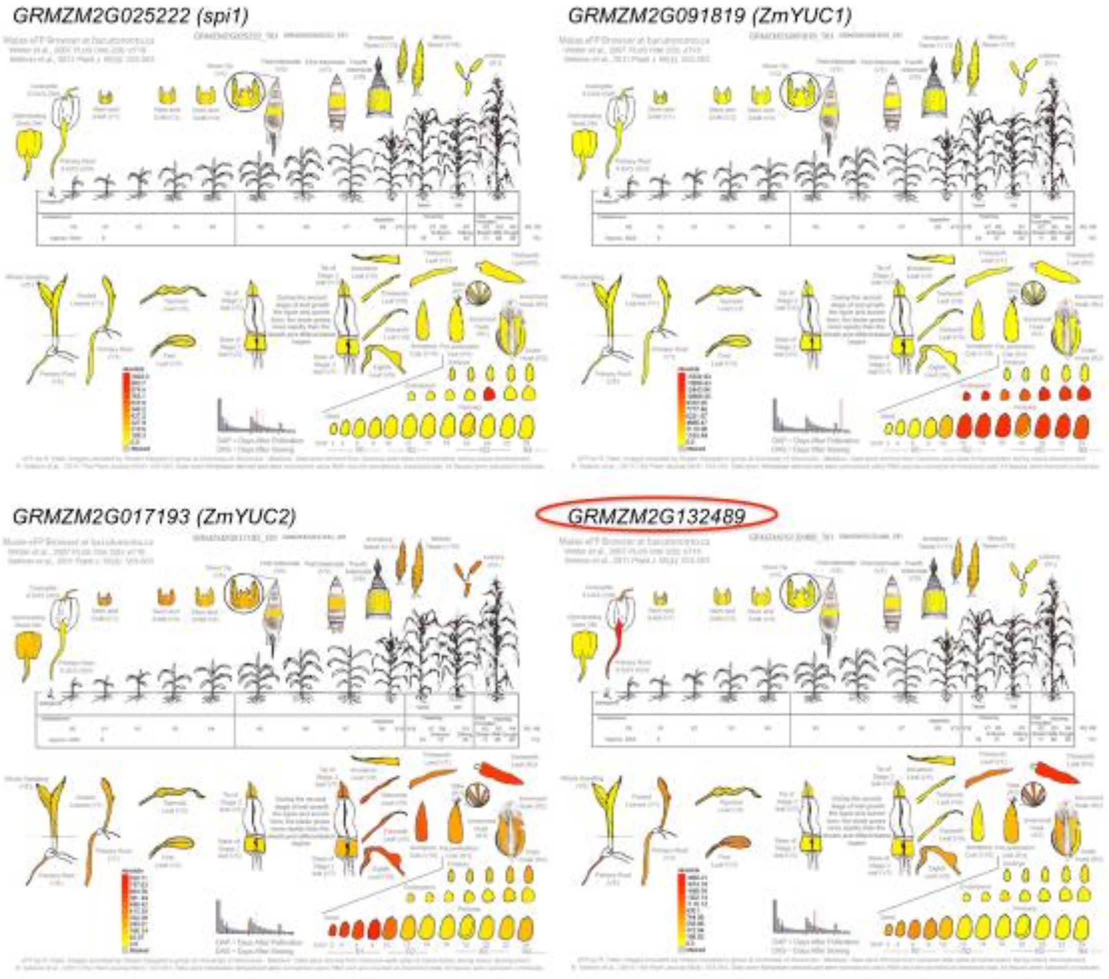
GRMZM2G025222 (spi1)
GRMZM2G091819 (ZmYUC1)
GRMZM2G017193 (ZmYUC2)
GRMZM2G132489

Figure S2.

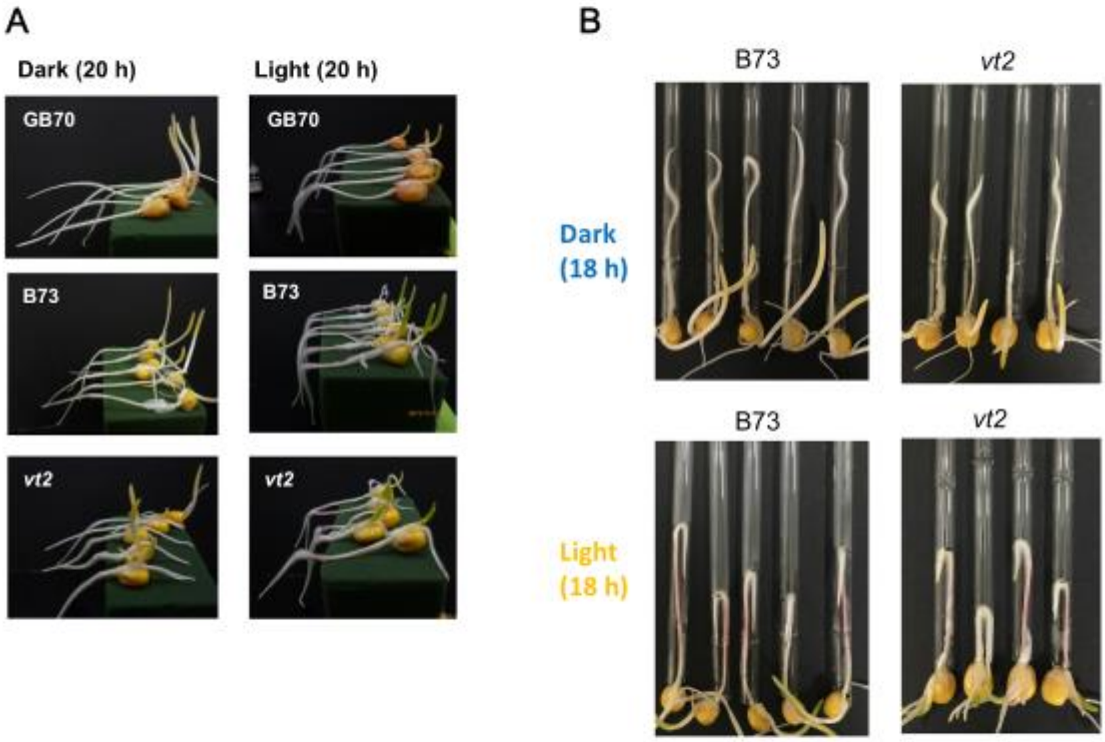
A
Dark (20 h)
Light (20 h)
GB70
GB70
B73
B73
vt2
vt2
B
B73
vt2
Dark
(18 h)
B73
vt2
Light
(18 h)

Figure S3 (1).

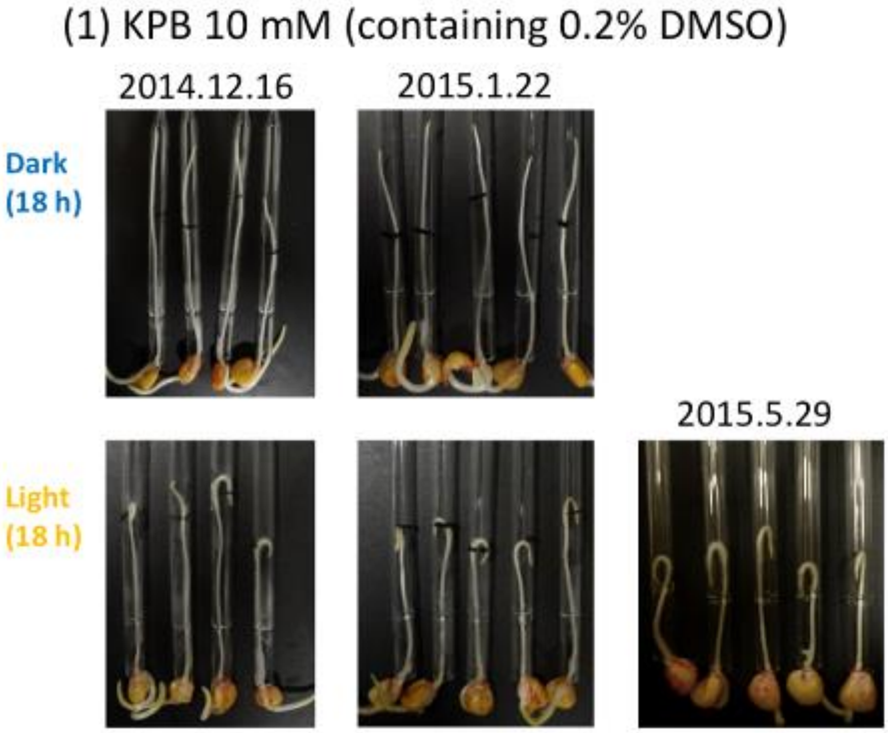
(1) KPB 10 mM (containing 0.2% DMSO)
2014.12.16
2015.1.22
2015.5.29
Dark
(18 h)
Light
(18 h)

Figure S3 (2).

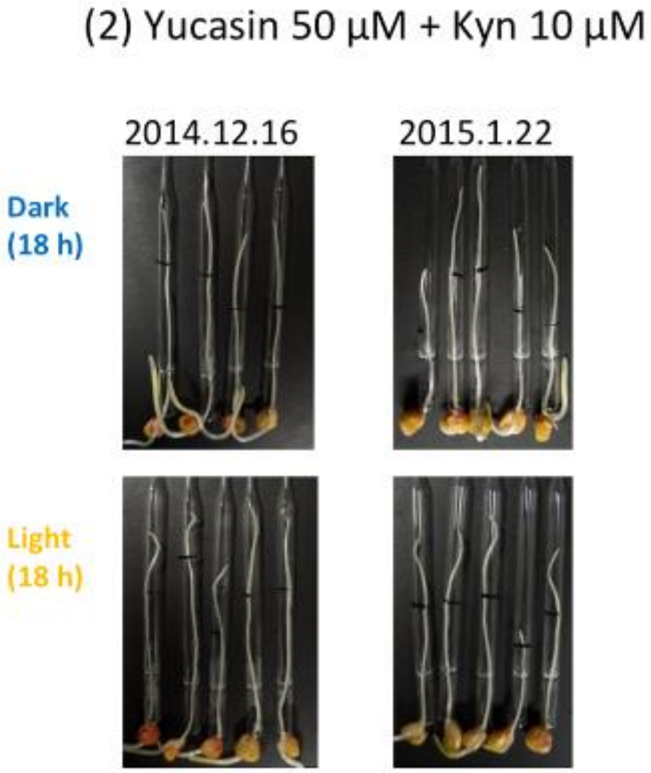

Figure S3 (3).

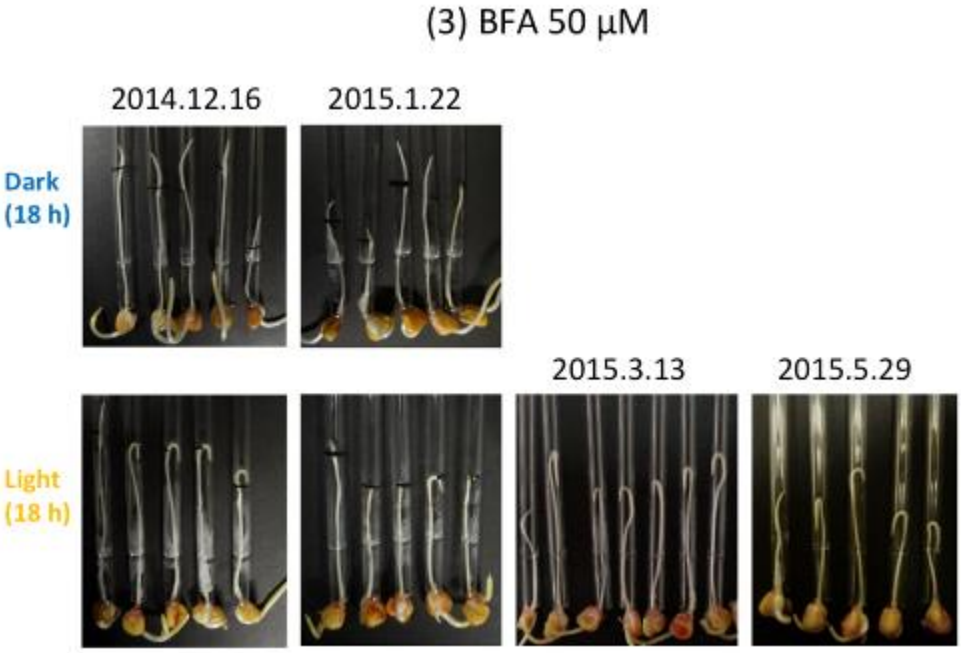
(3) BFA 50 μM
2014.12.16
2015.1.22
Dark
(18 h)
2015.3.13
2015.5.29
Light
(18 h)

Figure S3 (4).

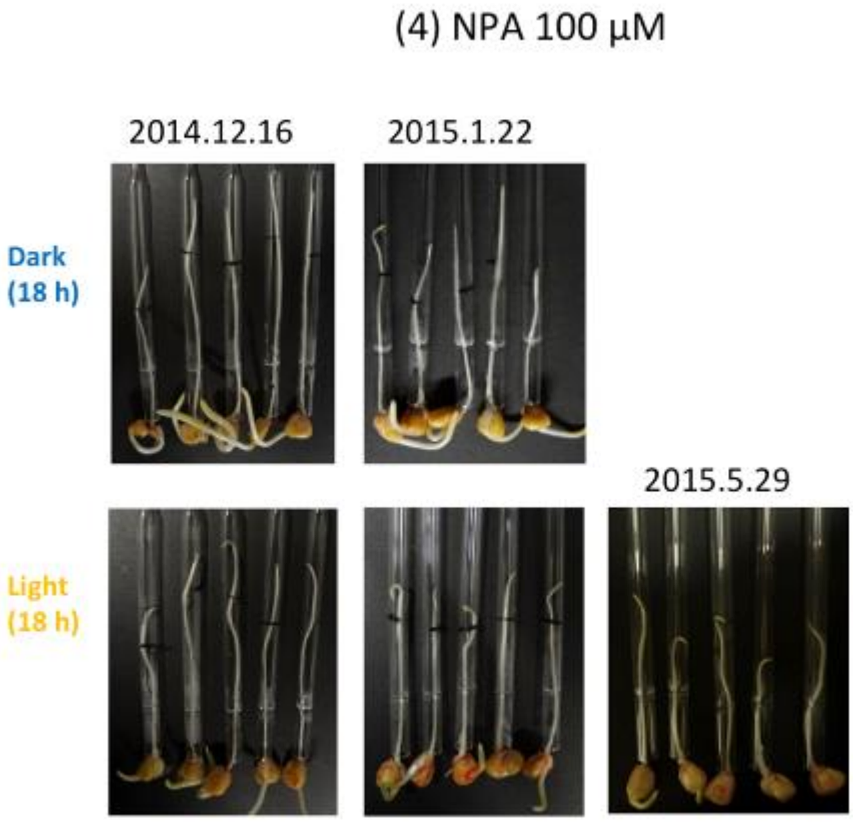

Figure S3 (5).

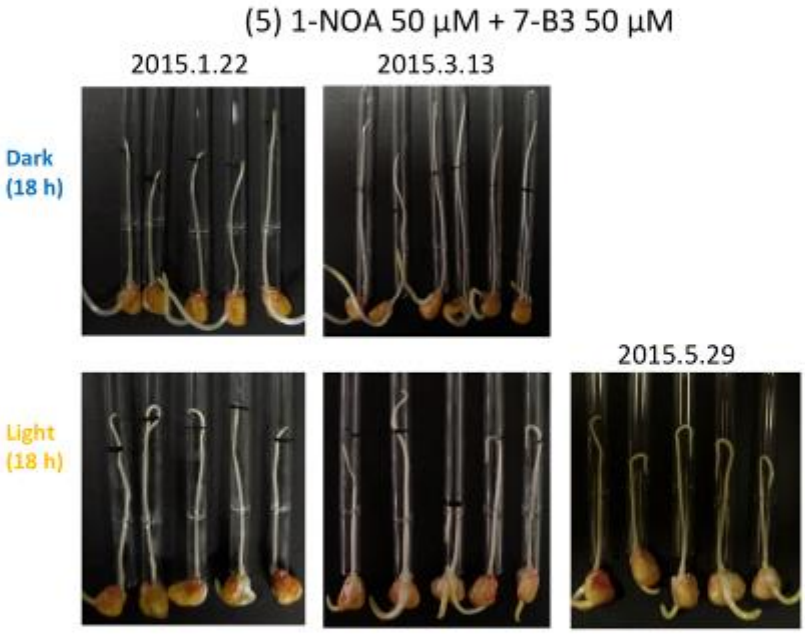
(5) 1-NOA 50 μM + 7-B3 50 μM
2015.1.22
2015.3.13
Dark
(18 h)
2015.5.29
Light
(18 h)

Figure S4.

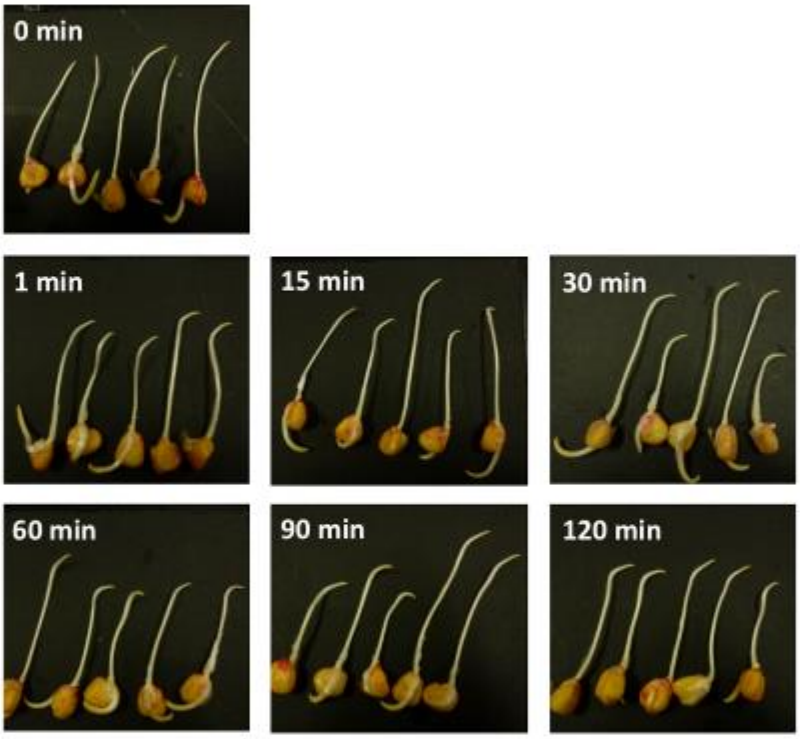

Figure S5.

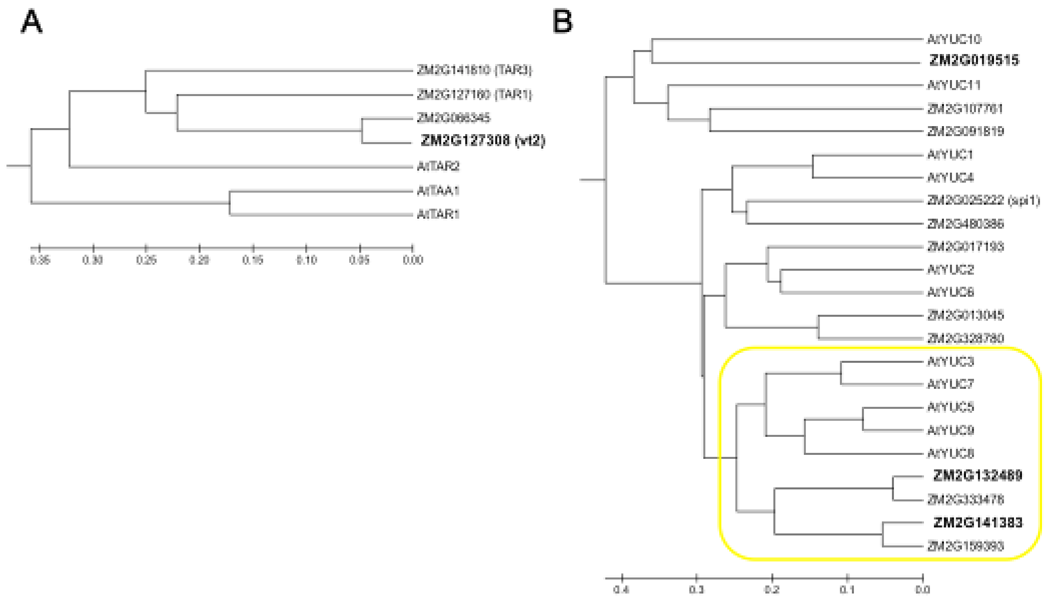
A
ZM2G141810 (TAR3)
ZM2G127160 (TAR1)
ZM2G066345
ZM2G127308 (vt2)
AtTAR2
AtTAA1
AtTAR1
0.35
0.30
0.25
0.20
0.15
0.10
0.05
0.00
B
AtYUC10
ZM2G019515
AtYUC11
ZM2G107761
ZM2G091819
AtYUC1
AtYUC4
ZM2G025222 (spi1)
ZM2G480386
ZM2G017193
AtYUC2
AtYUC6
ZM2G013045
ZM2G328780
AtYUC3
AtYUC7
AtYUC5
AtYUC9
AtYUC8
ZM2G132489
ZM2G333476
ZM2G141383
ZM2G159393
0.4
0.3
0.2
0.1
0.0

Table S1.

**Trp aminotransferase related genes**		
ID	Arabidopsis best hit (from Maize GDB)	Root-expressing genes (from eFP browser)
GRMZM2G127308 (vt2)	*AtTAR2*	+++
GRMZM2G127160 (TAR1)	*AtTAR2*	
GRMZM2G066345	*AtTAR2*	++
GRMZM2G141810 (TAR3)	*AtTAR2*	

**YUCCA genes**		
ID	Arabidopsis best hit (from Maize GDB)	
GRMZM2G013045	*AtYUC2*	+++
GRMZM2G019515	*AtYUC10*	+
GRMZM2G107761	*AtYUC10*	
GRMZM2G141383	*AtYUC8*	+++
GRMZM2G159393	*AtYUC8*	+++
GRMZM2G328780	*AtYUC6*	
GRMZM2G333478	*AtYUC3*	+++
GRMZM2G480386	*AtYUC8*	++
GRMZM2G025222 (spi1)	*AtYUC1*	
GRMZM2G091819	*AtYUC11*	
GRMZM2G017193	*AtYUC2*	
GRMZM2G132489	*AtYUC3*	+++

Table S2.

ID	Forward primer	Reverse primer
GRMZM2G127308 (vt2)	GCAGCACTAAGCTTAGCTTAGCT	CTCAGCTGAACAGCAGCAGT
GRMZM2G066345	TGCATCTAGATGCTGGCGC	CGTGATCGAGATTGATCACCGA
GRMZM2G013045	CCGGCCGAGATCATGGAC	GCTCACCGACGGGGAAG
GRMZM2G019515	CTGGAGCGCGACGACTG	GTACTTCTTGGTGAGGTGGAGG
GRMZM2G141383	TGGGGCTCCTCCCTACC	GCTCCAGGACGACGAACG
GRMZM2G159393	AAGTCCACCTTCCAGCTCGC	GCCGAGCTTGGCGAGGT
GRMZM2G333478	CAGTTCGTGGAGTACCTGCAG	CTATGTACTCGGTGGTGGAAGTG
GRMZM2G480386	ACGACGACTGAGACCGAGT	CCTCATGCCCTTGAACCTCTC
GRMZM2G132489	GACCTCTGCGACCACAATG	AGGAAGCGGAGCAGGAAG
